# Stabilization of Positive Linear Discrete-Time Systems by Using a Brauer's Theorem

**DOI:** 10.1155/2014/856356

**Published:** 2014-08-11

**Authors:** Begoña Cantó, Rafael Cantó, Snezhana Kostova

**Affiliations:** ^1^Institut de Matemàtica Multidisciplinar, Universitat Politècnica de València, 46071 València, Spain; ^2^Institute of System Engineering and Robotics, Bulgarian Academy of Sciences, 1113 Sofia, Bulgaria

## Abstract

The stabilization problem of positive linear discrete-time systems (PLDS) by linear state feedback is considered. A method based on a Brauer's theorem is proposed for solving the problem. It allows us to modify some eigenvalues of the system without changing the rest of them. The problem is studied for the single-input single-output (SISO) and for multi-input multioutput (MIMO) cases and sufficient conditions for stability and positivity of the closed-loop system are proved. The results are illustrated by numerical examples and the proposed method is used in stochastic systems.

## 1. Introduction

Positive systems are used to model many applications fields such as biology, chemistry, ecology, economy, and sociology (see [1] and the references therein). These systems have the peculiar property that any nonnegative input and nonnegative initial state generate a nonnegative state trajectory and a nonnegative output for all time. The positivity of the variables often emerges as the immediate consequence of the nature of modeled process, such as any variable representing a different type of resource like time, money and goods, buffer size and queues, data packets flowing in a network, water and air flows, populations, concentration of any substance, electric charge, and light intensity levels. For a good introduction to the theory of positive systems see [2, 3].

Many well-established results for general linear systems cannot be directly applied to positive systems. This feature makes the study of positive systems very interesting and many results have been obtained in this area both for continuous and for discrete-time systems [4, 5].

The stability property is fundamental to the correct functioning of any control system and in particular of positive control systems. Stabilization of linear systems using feedback has attracted considerable interest during the last decades. Various approaches have been used to study the aspects of the stabilization problem, namely, the condition under which the linear system described in the state-space can be stabilized via feedback. For instance, some fundamental results on stability property of linear systems are given in [6–8]. When considering the stabilization problem of positive systems, the additional conditions exist on the feedback, ensuring that the closed system remains positive. In this case, it is possible that the generated feedback can be entrywise nonpositive. For example, in biology a nonnegative input means that the species can never be killed, and this situation is not realistic [9].

This problem has been studied by several authors, but it is not completely solved yet. For instance, in [10] it is considered for discrete-time periodic linear systems and in [11] for positive switched systems.

The goal of this paper is to propose a new method for stabilization of positive unstable linear discrete-time systems, maintaining its positivity, that is, to use linear state feedback such that the eigenvalues of the closed-loop system have magnitude less than one. For that, we use a Brauer's theorem to modify some eigenvalues of the state matrix without changing any of the remaining eigenvalues (see [12, 13] and the references given there).

The obtained results on stability property are applied to stochastic matrices to construct a closed-loop system whose eigenvalues are less than one and it maintains the positivity. Stochastic systems are included in systems theory that deals with dynamic as well as static systems, whose processes are characterized by probability distributions or spectral measures. These systems can be modeled by a discrete-time system where the matrix *A* has a stochastic structure and it can be used, for example, to model the evolution of nondeterministic events. Moreover, it is known that stochastic matrices play an important role in economic models; see [14]. Some results concerning eigenvalues of stochastic matrices and their applications to nonnegative matrices are presented in [15]. The development of the stochastic stability theory is based on Markov parameters and on a Lyapunov approach (see more information in [16] and references therein).

The paper is organized as follows. In Section 2 we present the main results for the stability and the positivity of the single-input single-output (SISO) system. In Section 3 we extend the SISO results for a particular multi-input multioutput (MIMO) system. In Section 4 we show an application to stochastic systems. Finally, in Section 5 concluding remarks and some perspectives are given.

## 2. Stabilization of SISO PLD System

We consider a SISO positive linear discrete-time system

(1) $$\begin{array}{c} x\left(k+1\right)=Ax\left(k\right)+bu\left(k\right),\;k\in Z_{+}, \end{array}$$

where *x*(0) ≥ 0, *A* = (*a*_*ij*_) ∈ *R*_+_^*n*×*n*^, *b* = (*b*_*i*_) ∈ *R*_+_^*n*×1^, *x*(*k*) is the state vector, and *u*(*k*) is the control vector. This system can be represented by the pair of matrices (*A*, *b*).

It is known that the linear discrete-time system (1) is asymptotically stable if and only if the dominant eigenvalue *ρ* = *ρ*(*A*) is smaller than 1. If *ρ* > 1, then the system is unstable and if *ρ* = 1 the system is said to be marginally stable.

We consider a positive system (1) with *ρ* ≥ 1. Our goal is to construct a state feedback vector *f* ∈ *R* ^*n*×1^ such that *u*(*k*) = *f* ^*T*^ *x*(*k*) and the closed-loop system

(2) $$\begin{array}{c} x\left(k+1\right)=\left(A+bf^{T}\right)x\left(k\right)=A_{c}x\left(k\right) \end{array}$$

is positive; that is, *A*_*c*_ ≥ 0 and is asymptotically stable; that is, *ρ*(*A*_*c*_) < 1.

For solving the above problem, we use Brauer's theorem [17] that shows how to modify one single eigenvalue of *A* using a rank-one perturbation without changing any of the remaining eigenvalues.

**Theorem 1 (see [12, 13, 17, 18]).**

Let *A* be an *n* × *n* arbitrary matrix with eigenvalues *σ*(*A*) = {*λ*_1_, *λ*_2_,…, *λ*_*n*_}. Let *x*_*k*_ be an eigenvector of *A* associated with the eigenvalue *λ*_*k*_, and let *q* be any *n*-dimensional vector. Then the matrix *A* + *x*_*k*_ *q* ^*T*^ has eigenvalues {*λ*_1_,…, *λ*_*k*−1_, *λ*_*k*_ + *x*_*k*_^*T*^ *q*, *λ*_*k*+1_,…, *λ*_*n*_}.

**Remark 2 .**

Note that if *x*_*k*_^*T*^ *q* = 0 then the eigenvalues of the updated matrix *A* + *x*_*k*_ *q* ^*T*^ are the same as the initial matrix *A*.

In [12, 13, 18] several applications of the above theorem are presented concerning the stabilization, nonnegative inverse eigenvalue, and pole assignment problems for SISO systems.

**Proposition 3 (see [12, proposition 2.4]).**

Consider the pair (*A*, *b*) that represents a single-input single-output linear time invariant control system. Let *σ*(*A*) = {*λ*_1_, *λ*_2_,…, *λ*_*n*_} and let *x*_*k*_ be an eigenvector of *A* ^*T*^ associated with *λ*_*k*_. If *b* ^*T*^ *x*_*k*_ ≠ 0, then there exists a vector *f* such that *σ*(*A* + *bf* ^*T*^) = {*λ*_1_,…, *λ*_*k*−1_, *λ*_*k*_ + *x*_*k*_^*T*^ *b*, *λ*_*k*+1_,…, *λ*_*n*_}.

**Remark 4 .**

By Remark 2, if *x*_*k*_^*T*^ *b* = 0 then the eigenvalues do not change and the closed-loop system with *A*_*c*_ = *A* + *bf* ^*T*^ has the same stability property as the initial system (1).

From the theory of nonnegative matrices [19], the Perron-Frobenius theorem states that if *A* (the same for *A* ^*T*^) is a nonnegative matrix, then it has a nonnegative eigenvalue *ρ* = *ρ*(*A*) = *ρ*(*A* ^*T*^), that is, the Perron root, which is greater than or equal to the modulus of each of the other eigenvalues, and its corresponding eigenvector *v* = *v*_*ρ*_ ∈ *R* ^*n*×1^, which is referred to as the Perron-Frobenius eigenvector of *A*, is also nonnegative. Furthermore, if *A* is irreducible then *ρ* is positive and the entries of *v* are strictly positive.

Next theorem studies the stabilization problem for a SISO PLD system (1) given by the pair (*A*, *b*). Note that *b* ^*T*^ *v* = *v* ^*T*^ *b* ≠ 0 is a consequence of Remark 4.

**Theorem 5 .**

Consider the pair (*A*, *b*) that represents a SISO PLD system. Let *σ*(*A*) = {*ρ*, *λ*_2_,…, *λ*_*n*_} and suppose that *ρ* ≥ 1 and |*λ*_*i*_ | <1, *i* = 2,…, *n*. Let *v* ≥ 0 be an eigenvector of *A* ^*T*^ associated with *ρ*. If

(a) $\hat{b}=b^{T}v\neq 0$,
(b) for each *a*_*ij*_ = 0 the corresponding *b*_*i*_ = 0, for *i*, *j* = 1,2,…, *n*,
(c) *α* ∈ ]*M*_1_, *M*_2_[ where
  (3) $$\begin{array}{c} M_{1}=\max\left\{\frac{-\rho }{\hat{b}},\underset{b_{i}v_{j}\neq 0}{\max}\left\{\frac{-a_{ij}}{b_{i}v_{j}}\right\}\right\},\;\;M_{2}=\frac{1-\rho }{\hat{b}}, \end{array}$$

then the control *u*(*k*) = *f* ^*T*^ *x*(*k*) with *f* = *αv* makes the closed-loop system positive and asymptotically stable.

**Proof**

Let $\mu =\rho +\alpha \hat{b}$, with $\hat{b}=b^{T}v\neq 0$, be the new eigenvalue of *A*_*c*_. From condition (c), if *α* > *M*_1_, it follows that $\alpha >-\rho /\hat{b}$; then *μ* > −1. Thus, the closed-loop system is asymptotically stable.

Now we will prove the positiveness of the system. The zero entries of *A* do not change by condition (b). As *α* > *M*_1_,

(4) $$\begin{array}{c} \alpha >\underset{b_{i}v_{j}\neq 0}{\max}\left\{\frac{-a_{ij}}{b_{i}v_{j}}\right\} \end{array}$$

and this implies that for every *h* and *k* such that *b*_*h*_ *v*_*k*_ ≠ 0 (and hence they are both positive) we have

(5) ahk+bhαvx>ahk+bhmax⁡bivj≠0{−aijbivj}vk≥ahk+bh{−ahkbhvk}vk=0.

This proves that *a*_*hk*_ + *b*_*h*_ *αv*_*x*_ is a positive entry because *b*_*h*_ and *v*_*k*_ are both positive. On the other hand, if either *b*_*h*_ or *v*_*k*_ is zero, then *a*_*hk*_ + *b*_*h*_ *αv*_*x*_ = *a*_*hk*_ ≥ 0.

Therefore, the closed-loop system is positive and asymptotically stable.

**Remark 6 .**

The assumption that *b* has a zero entry in every position in which *A* has a row containing a zero entry means that in this position the matrix *A* is not perturbed and its values are not modified. An application is given in [20].

**Remark 7 .**

If the matrix *A* is irreducible the condition (a) from Theorem 5 is unnecessary since, for *v* > 0 and *b* ≥ 0, $\hat{b}=b^{T}v$ is always different from 0.

By Theorem 5 we introduce the next procedure to obtain the value of *α* and the corresponding feedback vector *f* = *αv*.

*Procedure Input: *(*A* ^*T*^, *b*).

1. Obtain the spectral radius *ρ* of *A* ^*T*^ and let *v* be a nonnegative eigenvector of *A* ^*T*^ associated with *ρ*.
2. If *ρ* < 1, then the pair (*A*, *b*) is asymptotically stable.* END*.
3. Otherwise, if $\hat{b}=b^{T}v=0$, then this algorithm cannot be applied to stabilize the system.* END*.
4. For *i* = 1,2,…, *n*, check if *b*_*i*_ = 0 when there exists an element *a*_*ij*_ = 0 for *j* = 1,…, *n*. Otherwise, the feedback matrix *A*_*c*_ is not positive.* END*.
5. Calculate *d*_*ij*_ = −*a*_*ij*_/*b*_*i*_ *v*_*j*_ for all (*i*, *j*) such that *b*_*i*_ *v*_*j*_ ≠ 0. *D* = max⁡(*d*_*ij*_).
6. Calculate $E=-\rho /\hat{b}$. Consider *M*_1_ = max⁡(*D*, *E*), $M_{2}=\left(1-\rho \right)/\hat{b}$.
7. If *M*_1_ ≥ *M*_2_, then the closed-loop matrix *A*_*c*_ is not positive.* END*.
8. Otherwise, choose *α*∈]*M*_1_, *M*_2_[. Consider *f* = *αv* and *A*_*c*_ = *A* + *bf* ^*T*^.

**Example 8 .**

Consider the pair (*A*, *b*) that represents a SISO PLD system

(6) $$\begin{array}{c} A=\left(\begin{array}{ccc} 0.5 & 0 & 0.6\\ 0.6 & 0.8 & 1.2\\ 0.8 & 1 & 0.8 \end{array}\right),\;\;\;b=\left(\begin{array}{c} 0\\ 1\\ 1 \end{array}\right). \end{array}$$

Next, we follow the procedure as follows.

(1-2) Consider *σ*(*A*) = *σ*(*A* ^*T*^) = {2.1458,0.3542, −0.4}; then *ρ* = 2.1458 and an associated eigenvector *v* of *A* ^*T*^ is given by *v* = (0.7570,0.7430,1)^*T*^. Since *ρ* > 1, then the pair (*A*, *b*) is not asymptotically stable.
(3) Consider $\hat{b}=b^{T}v=1.7430\neq 0$; then the algorithm can be applied to stabilize the system.
(4)  For *a*_12_ = 0, *b*_1_ = 0.
(5-6) Consider *d*_21_ = −0.7926, *d*_22_ = −1.0767, *d*_23_ = −1.2, *d*_31_ = −1.0568, *d*_32_ = −1.3459, *d*_33_ = −0.8, *D* = *d*_21_ = −0.7926, *E* = −1.2311, *M*_1_ = −0.7926, and *M*_2_ = −0.6574.
(7-8) Since *M*_1_ < *M*_2_, *α*∈] − 0.7926, −0.6574[. Consider *f* = *αv* and *A*_*c*_ = *A* + *bf* ^*T*^.

Now we consider, for instance, *α* = −0.7. In this case

(7) $$\begin{array}{c} f={\left(-0.5299,-0.5201,-0.7\right)}^{T} \end{array}$$

and the closed-loop matrix is

(8) $$\begin{array}{c} A_{c}=\left(\begin{array}{ccc} 0.5 & 0 & 0.6\\ 0.0701 & 0.2799 & 0.5\\ 0.2701 & 0.4799 & 0.1 \end{array}\right)\geq 0, \end{array}$$

with *σ*(*A*_*c*_) = {0.9257,0.3542, −0.4}. Then, the closed-loop system is positive and asymptotically stable.

As it is seen from the above theorem and corresponding procedure we can select any value of the parameter *α* in the interval ]*M*_1_, *M*_2_[. The question which arises is how to use this freedom of choice to improve system behavior. In the context of our main goal to improve stability of the system, good idea is to choose parameter *α* in such way that it maximizes the stability radii of closed-loop system. The concept of stability radii is developed in [21, 22].

The stability radii measure the robustness of stability of the systems under perturbations with structure, defined by the matrices *D* and *E*, and it is defined by

(9) rK(A)=rK(A,D,E)=inf⁡{||Δ||:Δ∈Kl×q,ρ(A+DΔE)≥1},

where ||·|| is given operator norm and *K* = *R*, *C*.

If complex perturbations are allowed, the complex stability radius is obtained and is denoted by *r*_*C*_. If only real perturbations are considered the real stability radius is obtained and it is denoted by *r*_*R*_. The real and complex stability radii are in general distinct and their computation is difficult problem. Fortunately for positive systems the real and complex stability radii coincide (*r*_*R*_ = *r*_*C*_ = *r*) and they can be determined via an easy computable formula.

**Proposition 9 (see [23]).**

Suppose that (*A*, *D*, *E*) ∈ *R*_+_^*n*×*n*^ × *R*_+_^*q*×*l*^ × *R*_+_^*q*×*n*^, *ρ*(*A*) < 1, and *K* ^*l*^ and *K* ^*q*^ are provided with monotonic norms, *K* = *R* or *C*. Then

(10) rC(A;D;E)=rR(A;D;E)=r(A;D;E)=||G(1)||−1,

where *G*(*s*) = *E*(*sI* − *A*)^−1^ *D* and ||*G*(1)|| is the operator norm of *G*(1) : *R* ^*l*^ → *R* ^*q*^.

Let J* denotes the set of all state feedbacks preserving Shur stability and positivity of the closed-loop system with:

(11) $$\begin{array}{c} J^{*}=\left\{F\in R^{m\times n}:A+BF\geq 0,\rho \left(A+BF\right)<1\right\}. \end{array}$$

The following proposition is proved in [21].

**Proposition 10 .**

The map *r*(*F*) : *R* ^*m*×*n*^ → *R*_+_ is continuous and monotone on J*; that is, *F*_1_ ≤ *F*_2_⇒*r*(*F*_1_) ≥ *r*(*F*_2_).

Hence we can use freedom to choosee the parameter *α* to maximize the stability radii of the system; in other words, the closed-loop system matrix *A*_*c*_ will have minimal sensitivity to the affine system perturbation type *A* → *A*(Δ) = *A* + *D*Δ*E*. From Theorem 5 it is clear that the parameter *α* is negative and, according to Proposition 10, if we chose the parameter close to the left bound of the interval, the stability radii will be larger, the sensitivity of the system to perturbation will be better, and the robustness of the system will be guaranteed.

## 3. Stabilization of MIMO PLD System

In this section we consider the MIMO PLD system with only one eigenvalue greater than or equal to 1 and we apply the obtained result from SISO systems in this case. Let the MIMO PLD system

(12) $$\begin{array}{c} x\left(k+1\right)=Ax\left(k\right)+Bu\left(k\right),\;k\in Z_{+}, \end{array}$$

where *x*(0) ≥ 0, *A* = (*a*_*ij*_) ∈ *R*_+_^*n*×*n*^, and *B* = (*b*_*ij*_) ∈ *R*_+_^*n*×*m*^; *x*(*k*) is the state vector and *u*(*k*) is the control vector. This system can be represented by the pair of matrices (*A*, *B*).

We consider a positive system (12) with its spectral radius *ρ* = *ρ*(*A*) ≥ 1. Our goal is to construct a state feedback matrix *F* ∈ *R* ^*m*×*n*^ such that *u*(*k*) = *F* ^*T*^ *x*(*k*) and the closed-loop system

(13) $$\begin{array}{c} x\left(k+1\right)=\left(A+BF^{T}\right)x\left(k\right)=A_{c}x\left(k\right) \end{array}$$

is positive; that is, *A*_*c*_ ≥ 0 and is asymptotically stable; that is, *ρ*(*A*_*c*_) < 1.

From now on, we denote by *e*_*i*_ = (0,…, 0,1, 0,…, 0)^*T*^ ∈ *R* ^*m*×1^ the *i*th canonical vector with a 1 in the *i*th coordinate and 0's elsewhere. The next theorem follows directly from Theorem 5.

**Theorem 11 .**

Consider the pair (*A*, *B*) that represents a MIMO PLD system. Let *σ*(*A*) = {*ρ*, *λ*_2_,…, *λ*_*n*_} and suppose that *ρ* ≥ 1 and |*λ*_*i*_ | <1, *i* = 2,…, *n*. Let *x* ≥ 0 be an eigenvector of *A* ^*T*^ associated with *ρ*. Let *B*_*i*_ be the *i*th column of *B*, *i* = 1,2,…, *m*. If there exists at least one column *B*_*k*_ of *B* such that

(a) ${\hat{B}}_{k}=B_{k}^{T}v\neq 0$,
(b) for each *a*_*ij*_ = 0 the corresponding *b*_*ik*_ = 0, for *i*, *j* = 1,2,…, *n*,
(c) *α*_*k*_∈]*M*_1_, *M*_2_[, where
  (14) $$\begin{array}{c} M_{1}=\max\left\{\frac{-\rho }{{\hat{B}}_{k}},\underset{b_{ki}v_{j}\neq 0}{\max}\left\{\frac{-a_{ij}}{b_{ki}v_{j}}\right\}\right\},\;\;M_{2}=\frac{1-\rho }{{\hat{B}}_{k}}, \end{array}$$

then the control *u*(*k*) = *F* ^*T*^ *x*(*k*) with *F* = *α*_*k*_ *ve*_*k*_^*T*^, makes the closed-loop system positive and asymptotically stable.

**Example 12 .**

Consider the pair (*A*, *B*) that represents a MIMO PLD system

(15) A=(0.500.60.60.81.20.810.8),B=(010100.5110.8).

Note that *A* is the same as in Example 8. Now, we check the conditions of the theorem for each column of *B*.

(i)  For *B*_1_ we consider the results obtained in Example 8. Then *α*_1_∈] − 0.7926, −0.6574[:
  (16) F=α1ve1T=α1(0.75700.74301)(1,0,0)=α1(0.7570000.743000100)
  and *A*_*c*_ = *A* + *BF* ^*T*^, with *A*_*c*_ ≥ 0 and *ρ*(*A*_*c*_) < 1.
(ii) For *B*_2_, since *a*_12_ = 0 but *b*_12_ ≠ 0, then the procedure cannot be applied to stabilize the system using this column of *B*.
(iii) For *B*_3_, using the procedure for SISO PLD systems we obtain that *α*_3_∈] − 1, −0.9781[.

For this example, we conclude that there exist two columns of *B* such that the conditions of Theorem 11 are satisfied and different feedback matrices can be obtained for stabilization of the system in such way that positiveness is guaranteed.

## 4. Application: Stochastic Systems

Stochastic systems are becoming extensively used as realistic models of physical phenomena. They are at the core of a number of disciplines in engineering, social systems, markets, management actions, molecular biology, and epidemiology; see, for example, [24]. The theory of Markov processes comprises the largest and the most important part of the theory of stochastic processes. This is well recognized in the theory of queues and in branching processes [25].

Different problems concerning modeling, analysis, synthesis, and simulation of stochastic systems can be found in the literature. On stability property, in this section we apply the obtained results in previous sections to stochastic systems in order to obtain a closed-loop system asymptotically stable and positive.

For that, we consider the system (1) where *A* is a nonnegative left-stochastic matrix. A left stochastic matrix *A* is square with nonnegative elements satisfying

(17) $$\begin{array}{c} \overset{n}{\underset{i=1}{\sum }}a_{ij}=1,\;j=1,2,\ldots ,n. \end{array}$$

Note that the system is not asymptotically stable, because *ρ* = 1 is an eigenvalue of *A*. Thus, *σ*(*A*) = *σ*(*A* ^*T*^) = {1, *λ*_*i*_}, being |*λ*_*i*_ | <1, *i* = 2,3,…, *n*, and *v* = (1,1,…, 1)^*T*^ is an eigenvector of *A* ^*T*^ associated with *ρ* = 1.

The following result is a consequence of Theorem 5 where the condition (a) is fulfilled because $\hat{b}=b^{T}v=b_{1}+b_{2}+\cdots +b_{n}\neq 0$.

**Proposition 13 .**

Consider the pair (*A*, *b*) that represents a SISO PLD system, where *A* is a left-stochastic matrix. Let *σ*(*A*) = {1, *λ*_2_,…, *λ*_*n*_} and let *v* = (1,1,…, 1)^*T*^ be an eigenvector of *A* ^*T*^ associated with *ρ* = 1. If

 (b′) for each *a*_*ij*_ = 0 the corresponding *b*_*i*_ = 0, for *i*, *j* = 1,2,…, *n*,
 (c′) *α*∈]*M*, 0[, where
  (18) $$\begin{array}{c} M=\max\left\{\frac{-2}{\hat{b}},\underset{b_{i}\neq 0}{\max}\left\{\frac{-a_{ij}}{b_{i}}\right\}\right\}, \end{array}$$

then the feedback law *u*(*k*) = *f* ^*T*^ *x*(*k*) with *f* = *αv* makes the closed-loop system positive and asymptotically stable.

Note that the above proposition is useful when *A* is also a doubly stochastic matrix.

**Corollary 14 .**

For a MIMO PLD system (12), where *A* is a left-stochastic matrix or *A* is a double stochastic matrix with only one eigenvalue equal to 1, we combine the results given in Theorem 11 and Proposition 13 as the following examples show.

**Example 15 .**

Consider the pair (*A*, *b*) that represents a SISO PLD system, where *A* and *b* are given by

(19) $$\begin{array}{c} A=\left(\begin{array}{cccc} 0.1 & 0.2 & 0.3 & 0.4\\ 0.7 & 0 & 0 & 0.1\\ 0.2 & 0.8 & 0.2 & 0.3\\ 0 & 0 & 0.5 & 0.2 \end{array}\right),\;\;b=\left(\begin{array}{c} 0.1\\ 0\\ 0.3\\ 0 \end{array}\right). \end{array}$$

In this case *A* is a left-stochastic matrix and *σ*(*A*) = *σ*(*A* ^*T*^) = {1, −0.3454, −0.0773 + 0.4131*i*, −0.0773 − 0.4131*i*}.

Note that *a*_22_ = *a*_23_ = 0 and *b*_2_ = 0; and *a*_41_ = *a*_42_ = 0 and *b*_4_ = 0. That is, the condition (b′) of Proposition 13 is satisfied. Moreover, $\hat{b}=0.4$, $-2/\hat{b}=-5$ and max⁡{−*a*_*ij*_/*b*_*i*_} = −*a*_31_/*b*_3_ = −*a*_33_/*b*_3_ = −2/3 and

(20) $$\begin{array}{c} -\frac{2}{3}<\alpha <0. \end{array}$$

If, for instance, *α* = −0.5, then *f* = −0.5*e*,

(21) $$\begin{array}{c} A_{c}=A+bf^{T}=\left(\begin{array}{cccc} 0.05 & 0.15 & 0.25 & 0.35\\ 0.7 & 0 & 0 & 0.1\\ 0.05 & 0.65 & 0.05 & 0.15\\ 0 & 0 & 0.5 & 0.2 \end{array}\right)\geq 0, \end{array}$$

and *σ*(*A*_*c*_) = {0.8, −0.3454, −0.0773 + 0.4131*i*, −0.0773 − 0.4131*i*}. Then the closed-loop system is positive and asymptotically stable.

Now, we consider the system (1) where the doubly stochastic matrix *A* and the vector *b* are given by

(22) $$\begin{array}{c} A=\left(\begin{array}{ccc} 0.5 & 0.2 & 0.3\\ 0.5 & 0.4 & 0.1\\ 0 & 0.4 & 0.6 \end{array}\right),\;\;b=\left(\begin{array}{c} 0.1\\ 0.3\\ 0 \end{array}\right). \end{array}$$

We have *σ*(*A*) = *σ*(*A* ^*T*^) = {1,0.2500 + 0.1936*i*, 0.2500 − 0.1936*i*}. Then, the condition (b′) of Proposition 13 is satisfied. In this case, $\hat{b}=0.4$, $-2/\hat{b}=-5$, and max⁡{−*a*_*ij*_/*b*_*i*_} = −*a*_23_/*b*_2_ = −1/3. Then −1/3 < *α* < 0.

If we consider, for instance, *α* = −0.1, then *f* = −0.1*e*,

(23) $$\begin{array}{c} A_{c}=\left(\begin{array}{ccc} 0.49 & 0.19 & 0.29\\ 0.47 & 0.37 & 0.07\\ 0 & 0.4 & 0.6 \end{array}\right)\geq 0, \end{array}$$

and *σ*(*A*_*c*_) = {0.96,0.2500 + 0.1936*i*, 0.2500 − 0.1936*i*}. Then the closed-loop system is positive and asymptotically stable.

## 5. Conclusions

Motivated by results obtained in [12] concerning application of Brauer's theorem for solving several control problems, we have considered a stabilization problem for both SISO and MIMO positive linear discrete time system by using linear state feedback. We have given simple sufficient conditions for the system matrices ensuring solvability of the stabilization problem. In this paper we consider the case when the only spectral radius is greater or equal to 1. We have shown that several choices for feedback matrix are possible according to the parameter *α*. That fact gives us possibility to satisfy other requirements on system behavior. In the future the same problems will be solved for the case when more than one eigenvalue is greater than or equal to 1. In this case Rado's theorem will be used, which is extension of Brauer's theorem.
